# Damage Identification of Piles Based on Vibration Characteristics

**DOI:** 10.1155/2014/150516

**Published:** 2014-11-17

**Authors:** Xiaozhong Zhang, Wenjuan Yao, Bo Chen, Dewen Liu

**Affiliations:** ^1^Department of Civil Engineering, Shanghai University, Shanghai 200072, China; ^2^School of Architectural Engineering, Quzhou University, Quzhou, Zhejiang 324000, China; ^3^College of Civil Engineering Kunming, Southwest Forestry University, Yunnan 650000, China

## Abstract

A method of damage identification of piles was established by using vibration characteristics. The approach focused on the application of the element strain energy and sensitive modals. A damage identification equation of piles was deduced using the structural vibration equation. The equation contained three major factors: change rate of element modal strain energy, damage factor of pile, and sensitivity factor of modal damage. The sensitive modals of damage identification were selected by using sensitivity factor of modal damage firstly. Subsequently, the indexes for early-warning of pile damage were established by applying the change rate of strain energy. Then the technology of computational analysis of wavelet transform was used to damage identification for pile. The identification of small damage of pile was completely achieved, including the location of damage and the extent of damage. In the process of identifying the extent of damage of pile, the equation of damage identification was used in many times. Finally, a stadium project was used as an example to demonstrate the effectiveness of the proposed method of damage identification for piles. The correctness and practicability of the proposed method were verified by comparing the results of damage identification with that of low strain test. The research provided a new way for damage identification of piles.

## 1. Introduction

In practical engineering, the quality of piles was influenced by various factors, including the conditions of geotechnical engineering, structural design, construction quality, and the surrounding environment. As a result, the problems of pile's quality could not be easily found and the accidents were difficult to deal. Due to the imperceptibility and complexity of pile foundation, and the limitations of the existing identification methods, there are numerous veiled issues on theories and practices of pile damage detection [1–4]. Therefore, an early detection of the crack damage of pile foundation and quick formulation of relevant prevention measures has become challenging topics for each evaluator on structural health. Regarding the pile quality problems in the construction, a variety of detection technologies have been developed in the past 50 years. Chen [5] researched the application of core drilling method to test pile damage, but this method was not able to accurately judge the fracture and the development of piles foundation in service. Xiao [6] established the method of application using ultrasonic transmission method in detecting the defects of piles. Liu et al. [7] applied the low strain method to detect the defects of piles. Wu et al. [8] proposed an approach to test the damage of foundation piles with a combination of applications of core drilling method, ultrasonic transmission method, and low strain integrity testing. The demonstrated results verified the reliability of the technology to identify the damage of piles. The above studies are all related to bridges or buildings under construction. However, due to the restriction of superstructure of built bridges or buildings, the usual pile testing methods are not fully applicable to determine the integrality of pile foundation. Johnson and Rausche [9] and Luo et al. [10] successively proposed to use the low strain dual velocity method, ultraseismic method, and equal seismic methods in detecting damage of the pile foundation in service. In 1995, Japanese researchers explored to apply hole camera technology to roughly determine the degree of cracks and damage of the foundation piles in southern Hyogo of postearthquake period, but they did not achieve good results for various reasons [11]. In 2009, Yuan et al. [12] determined the integrality of the bridge piles in service by collecting displacement signals in the condition that horizontal impact load was integrated with static loading. Qi, in 2012, achieved accurate detection results by applying full-bore wall imaging technology in detecting damage of the pile foundation in service [13]. However, in this process, certain damage was occurred when the cores of piles were drilling. In order to weaken the influence on test signal of foundation pile, which was caused by flat-slab superstructure, Zhang and Tian [14] used wavelet analysis method to eliminate the interference signal and applied wavelet analysis technology to detect the integrality of high-pile.

Dong et al. [15] proposed a damage detection method based on both the changes in strain mode shape and changes in resonant frequency. The difference between the damaged and undamaged structures was calculated by considering the index Δ*ϕ*_*i*_ that is given by

(1) $$\begin{array}{c} \Delta \phi_{i}={\left(\frac{\omega_{i}^{u}}{\omega_{i}^{d}}\right)}^{2}\phi_{i}^{d}-\phi_{i}^{u}, \end{array}$$

where *ω*_*i*_^*u*^ and *ω*_*i*_^*d*^ are the pulsations of the *i*th mode for the undamaged and damaged structures. *ϕ*_*i*_^*u*^ and *ϕ*_*i*_^*d*^ are, respectively, the *i*th strain mode shape of the undamaged and damaged structures. It was demonstrated that the index Δ*ϕ*_*i*_ was more sensitive to the damage severity when compared with the similar index calculated by displacement eigen parameter.

Another damage detection method focused on the decrease of modal stain energy between two structural degrees of freedom. This technique was proposed by Stubbs et al. [16, 17]. For a Bernoulli-Euler beam, a damage index was given to identify the damage in beams.

The mode strain energy was performed by Parloo et al. [18] for the identification of various damages in the I-40 highway bridge in New Mexico. It was shown that the method was not capable of achieving an efficient and robust detection of small damage due to measurement noise.

Alvandi and Cremona [19] used the strain energy method for damage detection not only in beam but also in civil engineering structures with experimental data. They showed that the strain energy method presented the best stability against disturbing signals. Even if the strain energy method appeared to be more efficient than other tested methods in the complex and simultaneous damage case, they indicated that the detection of two damages in the structures can be more difficult and a specific procedure should be applied. The same conclusions were reported by authors if the damage was located near supports or joints.

Fan and Qiao [20] presented a strain energy based identification method to identify damage of plate-type structure. The proposed method was response-based identification technique which required modal frequencies and curvature mode shapes before and after damage. However, the bending stiffness of the elements in the structures was assumed to be constant in this study, while in other structures it will be no longer a constant in the real progress. So study on strain energy identification method should be a promising subject for the development of the real-time structural health monitoring system in the future.

Yao and Zhang [21] have established a sensitive identification method for fracture damage identification of piles. But they only proposed the method; theoretical derivation is not very tight and there is no test to verify. So in this paper, the theory of damage identification was derived rigorously on the basis of result of [21]. In addition to this, numerical methods and experimental approach were employed to verify the proposed method.

In summary, scientists have done a series of studies on structural damage detection with strain energy method, and they have achieved certain research results. However, regarding the damage detection in the pile foundation of the structure in service, the work is conducted in the exploratory stage, and arduous efforts and further researches are needed before it can be much useful in engineering practices. Particularly for the small initial crack damage detection of pile foundation, it needs further theoretical analysis and exploration in practice. Taking full advantages of the natural frequency on integral damage identification, the sensitivity of the element modal strain energy method on small injuries detection, and the efficient local analysis of Time domain and frequency domain of wavelet analysis, an identification method for locating the small injure of pile and measuring damage severity was established in this paper, of which the correctness and efficiency were also demonstrated by engineering application examples.

## 2. Basic Theory

### 2.1. Eigenvalue Equation of Structural Vibration with Fracture Damage

The general cause of fracture damage is the decrease in local stiffness of the structure. Therefore, according to the perturbation theory, the structural vibration eigenvalue equation is as follows [22]:

(2) $$\begin{array}{c} \left[\left(K+\Delta K\right)-\left(\lambda_{i}+\Delta \lambda_{i}\right)M\right]\left(\phi_{i}+\Delta \phi_{i}\right)=0, \end{array}$$

where

(3) ΔK=∑j=1nΔKj=−∑j=1nαjKj (0≤αj≤1)Δλi=Δωi2=ϕiTΔKϕi;

*M*, *K* represent, respectively, the mass matrix and stiffness matrix; *ϕ*_*i*_ represents the *i*th order modal vector; *λ*_*i*_ represents the eigenvalue of *i*th modal of the vibration system, *λ*_*i*_ = *ω*_*i*_^2^ = (2*πf*)^2^; Δ*K* represents changes of structural stiffness; *α*_*j*_ represents *j*th element damage factor of the structure; *K*_*j*_ represents structural stiffness matrix of the *j*th element before damage; *n* represents the total number of elements of the structure. Expand (2) and then finish structural vibration eigenvalue equation; (2) of the structure can be expressed as follows:

(4) $$\begin{array}{c} \phi_{i}^{T}\Delta K\phi_{i}+\delta_{i}\phi_{i}^{T}K\phi_{i}+\phi_{i}^{T}\Delta K\Delta \phi_{i}+\delta_{i}\phi_{i}^{T}K\Delta \phi_{i}=0, \end{array}$$

where *δ*_*i*_ = −Δ*λ*_*i*_/*λ*_*i*_ is change rate of structure eigenvalue, which is mainly used to determine sensitivity of the *i*th modal on structural damage detection. Therefore, it is called modal damage sensitivity factor.

### 2.2. Identification Equation of Structural Damage

According to the energy balance theory, the released elastic strain energy was changed into plastic strain energy and surface energy when pile cracks occur, which stimulates the crack propagation, thus causing the decrease in structural strain in the macroscopic level. As a result, (4) can be expressed in the form of the strain energy. For piles structures, define the *j*th element strain energy of *i*th modal as follows:

(5) MSEij=ϕiTKjϕi,MSEijd=ϕidTKjdϕid=1−αjϕidTKjϕid,

where the superscript “*d*” represents the damaged structure.

According to the above formulas we can get

(6) ϕiTKϕi=∑j=1nϕiTKjϕi=∑j=1nMSEij=MSEi,ϕiTΔKϕi=∑j=1nϕiTΔKjϕi=−∑j=1nαjϕiTKjϕi=−∑j=1nαjMSEij.

Ignore second-order or more high-end items; we can get

(7) ϕiTKΔϕi=∑j=1nϕiTKjΔϕi=∑j=1n[ϕidTKjϕid−ϕiTKjϕi+ϕidTΔKjϕid]=∑j=1n[(1−αj)ϕidTKjϕid−ϕiTKjϕi]=∑j=1n(MSEijd−MSEij)ϕiTΔKΔϕi=∑j=1nϕiTΔKjΔϕi=−∑j=1nαjϕiTKjΔϕi=−∑j=1nαj(MSEijd−MSEj).

Substitute (6) and (7) into (4), and (4) can be expressed as follows:

(8) $$\begin{array}{c} \delta_{i}\cdot \text{MS}{\text{E}}_{i}^{d}=\overset{n}{\underset{j=1}{\sum }}\left(\alpha_{j}\cdot \text{MS}{\text{E}}_{ij}^{d}\right), \end{array}$$

where MSE_*i*_^*d*^ = ∑_*j*=1_^*n*^MSE_*ij*_^*d*^ represent the total strain energy of the *i*th modal of injured structure.

Formula (8) is equation of structural damage identification, which is composed by modal damage sensitivity factor, modal strain energy of injured structure, and the element damage factor. Due to the sensitivity of strain energy on small damage [20], and the high accuracy and easy measurement of the modal damage sensitivity factor [23], (8) can be used to accurately identify the damage severity of structure's small injuries.

*δ*_*i*_ is the indicator used to determine the modal sensitivity on structural damage; the larger the value of *δ*_*i*_ is, the more sensitive the modal identification on structural damage is and vice versa. Therefore, we can select the modal for structural damage identification according to factor *δ*_*i*_, while, in practical application, a certain threshold value is set based on the environment and damage situation of the structure; the efficient mode appears when the actual threshold value is greater than the set one.

### 2.3. Calculation of Element Modal Strain Energy

For the pile structure, in the case that element is sufficiently small, element modal strain energy can be represented by the formula as follows:

(9) MSEij=ϕiTKjϕi=12∫bjbj+1EIj∂2ϕi∂z22dz,MSEijd=1−αjϕidTKjϕid=12∫bjbj+1EIjd∂2ϕid∂z22dz.

(EI)_*j*_ and (EI)_*j*_^*d*^ represent, respectively, the structural flexural rigidities of *j*th element before and after damage. *b*_*j*_ and *b*_*j*+1_ represent *z* coordinates of node *j* and node *j* + 1.

In experimental modal analysis, the flexural rigidity (EI)_*j*_^*d*^ is unknown, while, in the case of small injury, it can be substituted by flexural rigidity before injury ((EI)_*j*_). Considering that the selected elements are relatively small, so the flexural rigidity (EI)_*j*_ of the *j*th element can be approximated as a constant, which can be put outside of the integral sign. So (9) can be rewritten as follows:

(10) MSEij=12(EI)j∫bjbj+1∂2ϕi∂z22dz

(11) MSEijd=12(EI)j∫bjbj+1∂2ϕid∂z22dz

(∂^2^ *ϕ*_*i*_/∂*z* ^2^)^2^ in (11) can be replaced by the mean value of (*ϕ*_*ij*_′′)^2^ and (*ϕ*_*i*(*j*+1)_′′)^2^, so (11) can be expressed as follows:

(12) $$\begin{array}{c} \text{MS}{\text{E}}_{ij}=\frac{1}{4}{\left(\text{EI}\right)}_{j}\left[{\left(\phi_{ij}^{\prime \prime }\right)}^{2}+{\left(\phi_{i\left(j+1\right)}^{\prime \prime }\right)}^{2}\right]\left(b_{j}+b_{j+1}\right). \end{array}$$

Similarly, (13) can be rewritten as follows:

(13) $$\begin{array}{c} \text{MS}{\text{E}}_{ij}^{d}=\frac{1}{4}{\left(\text{EI}\right)}_{j}\left\{{\left[{\left(\phi_{ij}^{d}\right)}^{\prime \prime }\right]}^{2}+{\left[{\left(\phi_{i\left(j+1\right)}^{d}\right)}^{\prime \prime }\right]}^{2}\right\}\left(b_{j}+b_{j+1}\right), \end{array}$$

where (*ϕ*_*ij*_′′)^2^, (*ϕ*_*i*(*j*+1)_′′)^2^, [(*ϕ*_*ij*_^*d*^)′′]^2^, and [(*ϕ*_*i*(*j*+1)_^*d*^)′′]^2^ can be got by measuring displacement mode shapes.

### 2.4. Determination of Damage Location

The difference value between the element modal strain energies before and after damage will be set as the original signal of injury:

(14) $$\begin{array}{c} f_{j}\left(z\right)=\text{MS}{\text{E}}_{ij}^{d}-\text{MS}{\text{E}}_{ij}. \end{array}$$

The signal *f*_*j*_(*z*) was firstly fitted by cubic spline interpolation and then transformed by wavelet function for transform coefficients, which can be used to detect the damage location:

(15) $$\begin{array}{c} DI_{ij}=C{\left(a,b\right)}_{i,j}; \end{array}$$

*C*(*a*, *b*)_*i*,*j*_ represents the transformation coefficient of *j*th element, in which “*a*” is the scale parameter and “*b*” is the time parameter. *DI*_*ij*_ represents the location index of damaged element. So index *DI*_*ij*_ can be used to determine the damaged element; specifically, the larger value of index *DI*_*ij*_ is, the bigger possibility of a damaged *j*th element is.

In order to reduce the impact of random noise from the test mode shapes, multiorder efficient modals are used to diagnose structural damage location:

(16) $$\begin{array}{c} DI_{j}=\frac{1}{N}\sum_{m}DI_{ij}, \end{array}$$

where *N* is the number of selected efficient modals. In practical applications, a given threshold value of *DI*_*j*_ can be used to determine whether there is a damage or not in the structure.

### 2.5. Calculation of Damage Severity

After damaged element is determined, formula (8) will be applied to calculate the degree of element damage. The element damage factor *α*_*j*_ can be used to represent the damage severity. If there is only one location of damage, which is assumed in *k* element, then *α*_*j*_ = 0  (*j* ≠ *k*), only *α*_*k*_ ≠ 0. Equation (8) can be expressed:

(17) $$\begin{array}{c} \alpha_{k}=\frac{\delta_{i}\cdot \text{MS}{\text{E}}_{i}^{d}}{\text{MS}{\text{E}}_{ik}^{d}}. \end{array}$$

If there are multiple locations of damage, assuming that there are *m* elements (*k*_1_, *k*_2_,…, *k*_*m*_) damaged (*m* ≤ *n*), then *m* number of efficient modes (*q*_1_, *q*_2_,…, *q*_*m*_) are needed for the calculation. Therefore using the equation set, which consists of *m* equations, the corresponding damage factor *α* can be solved. The equation set is as follows:

(18) $$\begin{array}{c} \overset{-}{\delta }\cdot \text{MSED}=\overset{-}{\alpha }\cdot \text{MSEDD}, \end{array}$$

where

(19) δ−=δq1δq2δq3⋯δqm,MSED=MSEq1dMSEq2dMSEq3d⋮MSEqmd,α−=αk1αk2αk3⋯αkm,MSEDD =MSEq1k1MSEq2k1MSEq3k1⋯MSEqmk1MSEq1k2MSEq2k2MSEq3k2⋯MSEqmk2MSEq1k3MSEq2k3MSEq3k3⋯MSEqmk3⋮⋮⋮⋮⋮MSEq1kmMSEq2kmMSEq3km⋯MSEqmkm.

*p* represents the number of measured modals, *m* represents the number of damaged elements. If *p* < *m*, extended modal, which extracted by experimental modal expansion technique [24], can be applied in the calculation.

## 3. Example Verification

### 3.1. Overview of Project

**Figure 1:**
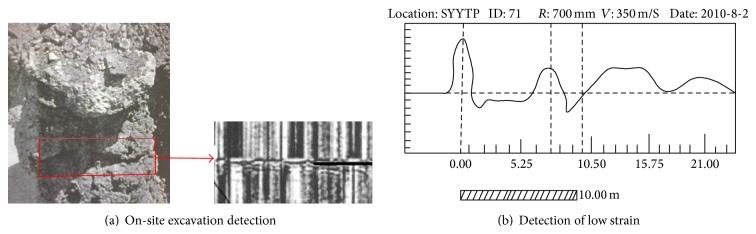
The actual damage of pile.

Natural hollowed filling piles and artificial hollowed filling piles were used in the foundation construction of Ouhai Sports Center Stadium with effective pile length ranging from 4 m to 30 m, diameter range from 600 mm to 1000 mm, and C25 of pile concrete strength. Totally there were 129 piles used in the project, according to the verification results of low strain detection and excavation; 9.6% of the damage occurred at 7.4 m from the top of number 71 pile. The length of number 71 pile is 21 m, and its diameter is 1 m. The actual damage of number 71 pile is shown in Figure 1.  Figure 1(b) is the ultrasonic strain curve of the pile.

### 3.2. Mode Measurement Using Top Acceleration and Deflections of the Pile

**Figure 2:**
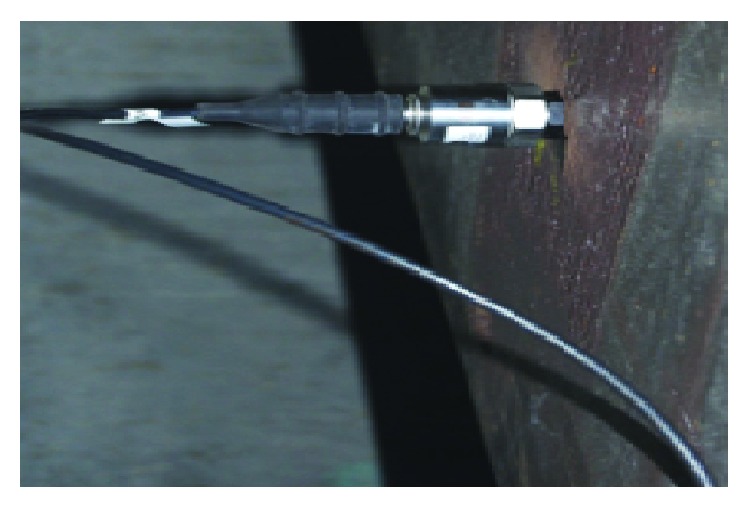
Typical accelerometer attachment.

**Figure 3:**
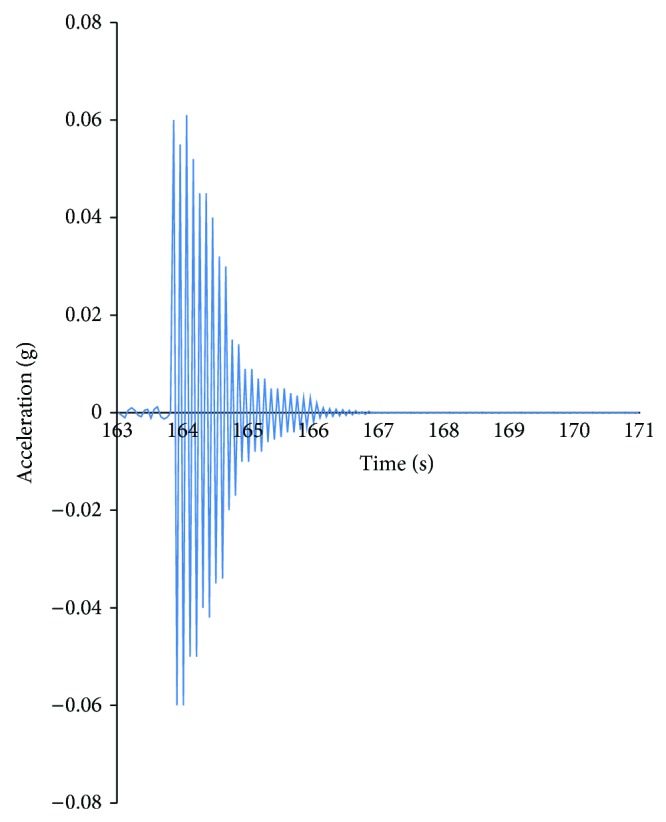
Acceleration response of sensor.

**Figure 4:**
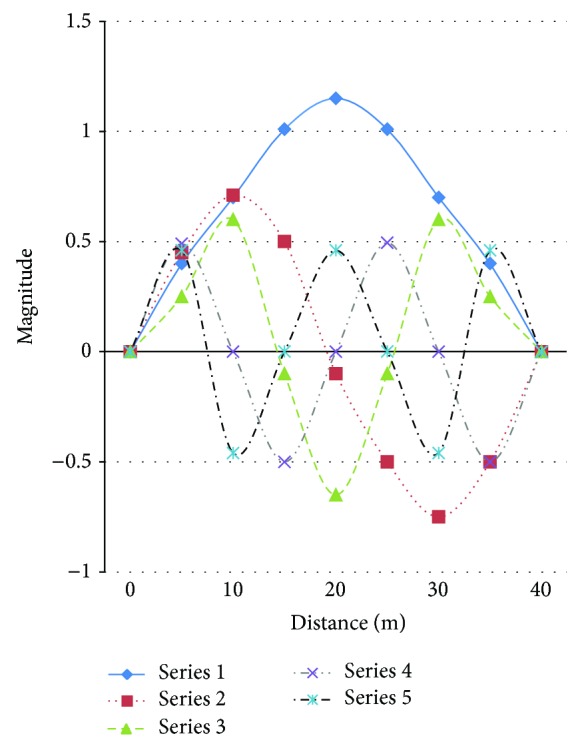
Mode shapes derived by sensors.

Typical accelerometer was attached on the structure at the top of the pile (Figure 2). The acceleration and deflection of the pile were obtained from the data collected by the accelerometer (Figure 3). Figure 3 is the acceleration response from 163 second to 171 second using ultrasonic technology.  Figure 4 is the top defection using displacement sensor.  Figure 4 shows the first five order modal shapes derived for sensors. Mode shapes (actual modal of the pile) and frequencies were derived by the acceleration and deflection (Table 1).

### 3.3. Finite Element Modeling

**Figure 5:**
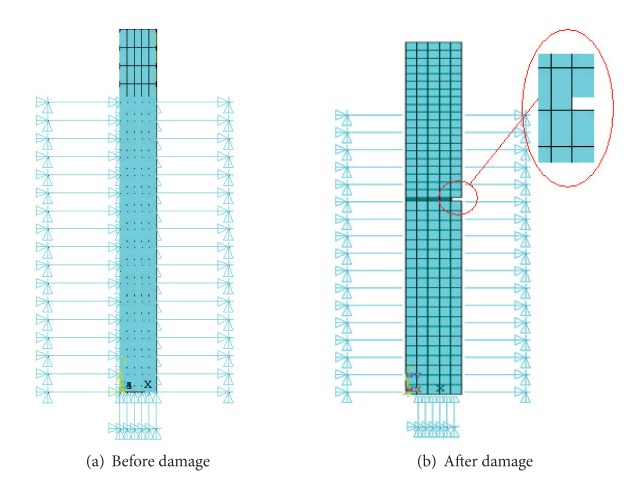
Finite element model of pile-soil interaction.

Applying finite element software ANSYS, we establish finite element model of soil-pile interaction for number 71 pile (Figure 5(a)) according to the design drawings. According to actual damage on the number 71 pile, the pile model of the cracks was established at 7.4 meters from the top of the pile with crack depth accounting for 9.6% of the diameter of pile foundation (Figure 5(b)). The element type of pile foundation model is SOLID45. The nonlinear spring element COMBIN139 is used to simulate pile-soil interaction. In order to establish a finite element computation model which is more consistent with the environment of actual engineering situation, the model updating technology was used to improve the finite element model based on the measured data.

### 3.4. Selection of Efficient Mode

**Figure 6:**
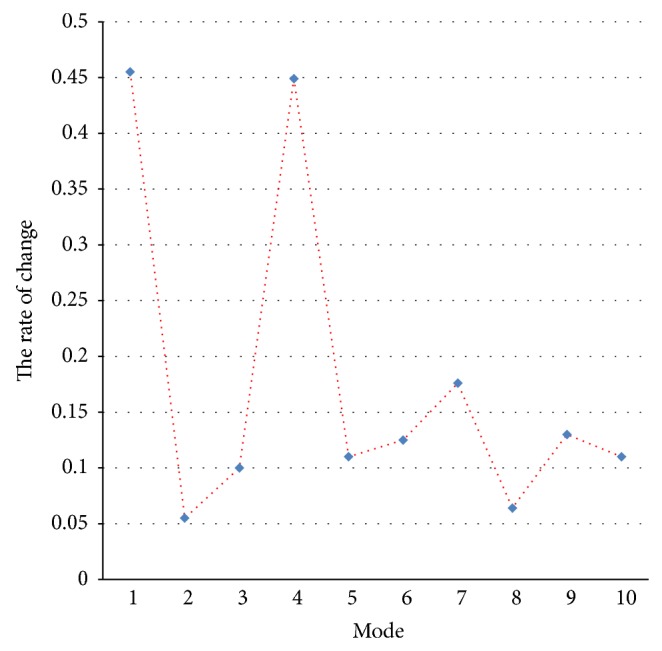
The change rate of the natural frequency.

The change rate of the natural frequencies before and after damage is shown in Figure 6 and Table 1, and the efficient frequency threshold is set as *δ*_*i*_ ≥ 0.4%. As Figure 6 shows, mode 1 and mode 4 are the efficient modes of damage case.

### 3.5. Damage Location Identification

**Figure 7:**
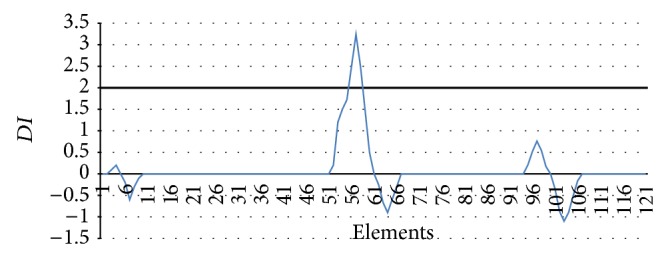
The DI of each element calculated by efficient modal.

Information function is established using the changes of element strain energy before and after damage, and wavelet transform is used to determine the parameters (*DI*) for the damage location. Efficient modal *DI*_*j*_ is shown in Figure 7. By setting the threshold (*DI* > 2), it can be clearly determined that crack damage occurred in the elements from 55 to 59, which is very identical with the actual damage location. The identification results of damage location are shown in tables. The identification error is 3%, which proves the accuracy advantage of damage location identification method.

### 3.6. Identification of Injury Severity

Element damage factor *α* is calculated by applying (18), and the results are shown in Table 2. As shown in Table 2, the result of actual injury is very close to that of calculated injury with identification error by 6.2%, which proves that the method of damage severity identification has a higher accuracy.

## 4. Conclusions

This paper firstly selected efficient modes for damage identification by applying the change rate of eigenvalues, and then it established an accurate method for small damage identification based on the application of sensitivity of element modal strain energy to small structural injury and efficient local analysis capabilities of wavelet analysis. The efficiency and accuracy of the proposed method were verified through identifying small cracks damage of the pile used in the Ouhai Sports Center. The conclusions are as follows.

1. Fracture identification method proposed in this paper can effectively and accurately identify the fracture damage location for the hidden structures in service.
2. The proposed method can effectively identify and quantify the fracture damage severity for the hidden structures in service, providing an important reference for the assessment of fracture damage.
3. The method presented in this paper provided a new approach for damage identification of concealed structure.

## Figures and Tables

**Table 1:** Calculated natural frequencies from the FE model.

Mode	The experimental monitoring frequencies	The calculated natural frequencies	Difference (%)
1	1.940	1.932	0.41
2	3.035	2.997	1.25
3	6.835	6.805	0.44
4	6.983	6.944	0.56
5	7.883	7.852	0.39

**Table 2:** Results of damage identification.

	Actual injury	Low strain method/error	New method/error
Damage location	7.4 m	6.71 m/9.3%	7.08 m/4.3%
Damage severity	9.6%	Small damage	10.2%/6.2%
